# Developing Messaging Content for a Physical Activity Smartphone App Tailored to Low-Income Patients: User-Centered Design and Crowdsourcing Approach

**DOI:** 10.2196/21177

**Published:** 2021-05-19

**Authors:** Laura Elizabeth Pathak, Adrian Aguilera, Joseph Jay Williams, Courtney Rees Lyles, Rosa Hernandez-Ramos, Jose Miramontes, Anupama Gunshekar Cemballi, Caroline Astrid Figueroa

**Affiliations:** 1 School of Social Welfare University of California, Berkeley Berkeley, CA United States; 2 Center for Vulnerable Populations University of California, San Francisco San Francisco, CA United States; 3 Department of Psychiatry University of California, San Francisco San Francisco, CA United States; 4 Department of Computer Science University of Toronto Toronto, ON Canada; 5 Division of General Internal Medicine Zuckerberg San Francisco General Hospital San Francisco, CA United States

**Keywords:** user centered design, mHealth, text messaging, crowdsourcing, mobile phone

## Abstract

**Background:**

Text messaging interventions can be an effective and efficient way to improve health behavioral changes. However, most texting interventions are neither tested nor designed with diverse end users, which could reduce their impact, and there is limited evidence regarding the optimal design methodology of health text messages tailored to low-income, low–health literacy populations and non-English speakers.

**Objective:**

This study aims to combine participant feedback, crowdsourced data, and researcher expertise to develop motivational text messages in English and Spanish that will be used in a smartphone app–based texting intervention that seeks to encourage physical activity in low-income minority patients with diabetes diagnoses and depression symptoms.

**Methods:**

The design process consisted of 5 phases and was iterative in nature, given that the findings from each step informed the subsequent steps. First, we designed messages to increase physical activity based on the behavior change theory and knowledge from the available evidence. Second, using user-centered design methods, we refined these messages after a card sorting task and semistructured interviews (N=10) and evaluated their likeability during a usability testing phase of the app prototype (N=8). Third, the messages were tested by English- and Spanish-speaking participants on the Amazon Mechanical Turk (MTurk) crowdsourcing platform (N=134). Participants on MTurk were asked to categorize the messages into overarching theoretical categories based on the capability, opportunity, motivation, and behavior framework. Finally, each coauthor rated the messages for their overall quality from 1 to 5. All messages were written at a sixth-grade or lower reading level and culturally adapted and translated into neutral Spanish by bilingual research staff.

**Results:**

A total of 200 messages were iteratively refined according to the feedback from target users gathered through user-centered design methods, crowdsourced results of a categorization test, and an expert review. User feedback was leveraged to discard unappealing messages and edit the thematic aspects of messages that did not resonate well with the target users. Overall, 54 messages were sorted into the correct theoretical categories at least 50% of the time in the MTurk categorization tasks and were rated 3.5 or higher by the research team members. These were included in the final text message bank, resulting in 18 messages per motivational category.

**Conclusions:**

By using an iterative process of expert opinion, feedback from participants that were reflective of our target study population, crowdsourcing, and feedback from the research team, we were able to acquire valuable inputs for the design of motivational text messages developed in English and Spanish with a low literacy level to increase physical activity. We describe the design considerations and lessons learned for the text messaging development process and provide a novel, integrative framework for future developers of health text messaging interventions.

## Introduction

### Background

Depression and diabetes are both highly disabling and often comorbid diseases that disproportionately affect patients with a low-income and ethnic minority status. For instance, ethnic and racial minority patients generally show a higher prevalence of these diseases [1], lower treatment rates [2,3], and worse outcomes [4,5]. There is a need for the design of more effective self-management interventions that can target both these diseases and are affordable, acceptable, and tailored to vulnerable populations [6]. Promoting physical activity is a potentially effective strategy that has positive effects on both mental health, with a particularly strong effect on depression [7,8], and common chronic diseases such as diabetes [9].

Mobile phone SMS, or text messaging, interventions have shown great promise in helping individuals engage in healthy behaviors, including physical activity [10,11] and diabetes self-management [12-14]. Most individuals currently own a mobile phone. Smartphone usage across a wide range of demographic groups is high (around 81%) and is on the rise. For instance, the percentage of Black individuals (80%) and Latino individuals (79%) that own smartphones is now similar to that of White individuals (82%) [15]. Therefore, feedback and motivational text messages might be effective in helping individuals with depression and diabetes increase their levels of physical activity and improve their overall health.

However, there is limited evidence regarding the design process of text messages aimed at promoting behavioral changes [16]. Detailed accounts of complete content development processes, including formative research and pretesting methods, are underreported in the mobile health (mHealth) literature [17]. Among the published health text messaging studies that describe their content design processes, there is great variability in the methods utilized. Researchers have reported the development of messaging based on public health guidelines [18-21], health education curricula [14,22], theoretical models [16,18,20,21,23], findings from quantitative surveys or focus groups [16,18,19,21,23,24], evaluation by members of the research team or outside experts [16,18,20,22], and pilot testing within a subset of the target population [19,20,25]. Thus, this study aims to integrate various methods such as participant feedback, pilot testing, crowdsourcing, and expert knowledge.

Moreover, very little has been described about the design process of text messages tailored to low-income, low–health literacy populations and non-English speakers. However, the current consensus is that digital interventions should emphasize usability and engagement with content [26] and should be developed with users who are intended to benefit from the intervention [27]. This perspective is fundamental to user-centered design (UCD), a design approach derived from the multidisciplinary field of study of human-computer interaction. UCD entails the active participation of end users in product development to enhance the understanding of user and task requirements as well as the iteration of design and evaluation [28].

In the context of health text messaging development, a UCD approach consists of conducting preliminary research within the target population to inductively identify any barriers that they face and collect feedback about the content as it is being developed [21,29,30]. However, recruiting large groups of participants within patient populations to test the content is challenging. Online crowdsourcing platforms, such as Amazon’s Mechanical Turk (MTurk), are increasingly being used as a means of acquiring feedback from a relatively large pool of participants. However, relying solely on MTurk respondents might not be entirely feasible as these participants are not always reflective of typical patient populations. Therefore, specifically for research on the design of text messaging in underserved populations, an argument can be made to combine crowdsourcing with participant feedback.

### Objectives

Considering the limited literature available and the lack of consensus on best practices for designing a text messaging intervention, particularly for low-income Spanish-speaking populations, we aimed to develop messaging that was both evidence based and responsive to user feedback. Here, we describe the iterative design process of text messages for use within an adaptive smartphone app for low-income ethnic minority patients. We present a novel framework for health text messaging design, the theory-informed UCD framework, which integrates a UCD approach with crowdsourcing and expertise within the study team. In seeking to advance the body of knowledge of mHealth development methods, we provide recommendations and *lessons learned* from designing text messages in this population.

## Methods

### Overview

This text messaging design is part of a larger randomized clinical trial, the *Diabetes and Mental Health Adaptive Notification Tracking and Evaluation* (DIAMANTE) trial (NCT 03490253) [31]. This trial examines the effectiveness of a smartphone app that uses a reinforcement learning algorithm to predict the categories of text messages that are most effective in increasing a participant’s physical activity, given the participant’s contextual variables, such as previous physical activity, as well as demographic and clinical variables (eg, age, gender, and depression scores) [32]. Although we plan to report the effectiveness results of the trial in future studies, this paper explains the development process of the different categories of motivational text messages for increasing physical activity targeted to individuals with comorbid diabetes and depression. The text messages were developed in 5 phases that incorporated crowdsourced feedback, study team feedback, and UCD techniques (ie, card sorting, interviews, and surveys). The process was iterative in nature as the findings from each step informed the subsequent steps.

### Message Development Phases

**Figure 1:**
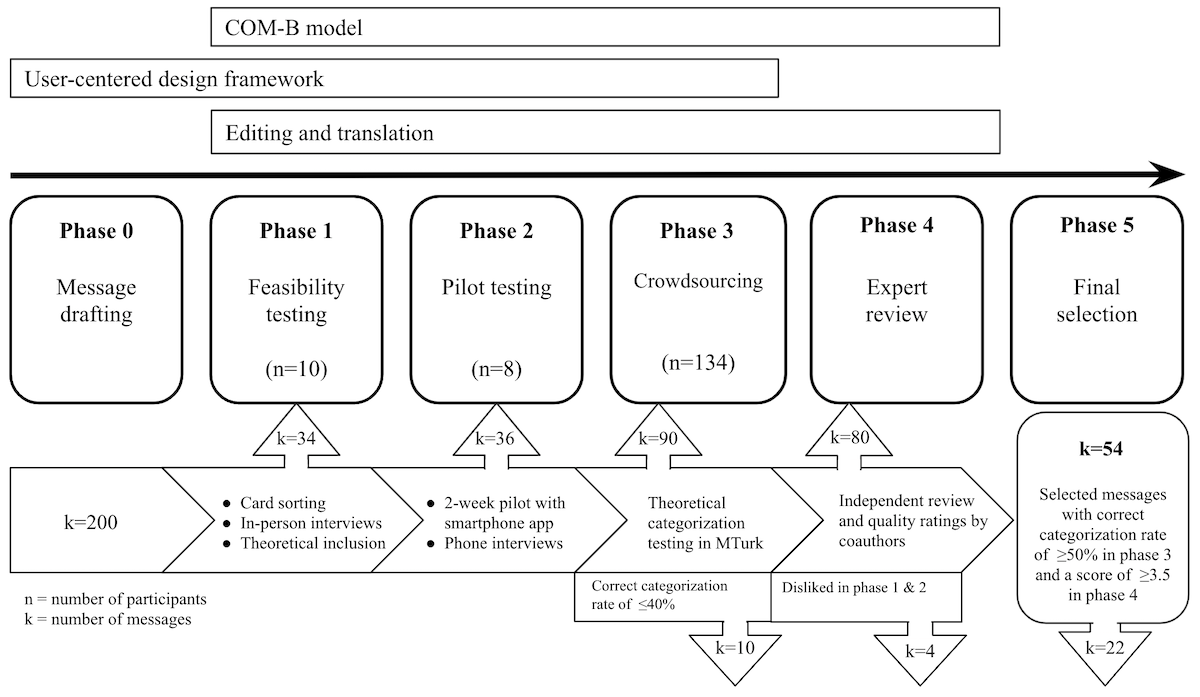
Theory-based, user-centered design framework: phases and methods of the message development process. COM-B: capability, opportunity, motivation, and behavior; MTurk: Amazon Mechanical Turk.

The messages were created in English and Spanish and tested in 5 phases (Figure 1).

#### Phase 0: Message Drafting

This phase occurred between August 2017 and June 2018. To ground our intervention in an understanding of the enablers and barriers to behavior change, we developed a library of concise messages (n=200) based on health behavior change constructs from social cognitive theory and evidence-informed guidelines for mHealth interventions found in the literature (eg, *gain and loss* framing, proximal outcomes framing) [33-37].

#### Phase 1: Feasibility Testing

This phase took place between July and December 2018. It consisted of a feasibility study phase with English-speaking (n=5) and Spanish-speaking (n=5) primary care adult patients recruited from a safety-net health care setting. All participants (10/10, 100%) were diagnosed with depression, and 90% (9/10) had diabetes [30,38]. A subset of messages (n=34) was tested using a mixed methods approach that included a card sorting task and individual semistructured interviews. For the card sorting activity, participants were asked to sort text messages in either English or Spanish (depending on their preferred language) into 3 piles: liked messages, disliked messages, and messages that were neither disliked nor liked (neutral). Participants also provided the reason behind the placement of the card in a particular pile. Although card sorting has been previously used to validate expert-developed health text messages among a group of nonexperts (ie, university students and staff) [39], the use of this method in our study entailed modification from the traditional card sorting protocol to adapt it to our population of low-income patients [40]. For example, the subset of messages represented in the cards was written at a sixth-grade reading level. In addition, interviewers modified the administration of the activity to accommodate participants with limited literacy or communication barriers by providing audiovisual cues, including reading the cards aloud and successively probing for feedback after reading each card [40].

The individual semistructured interviews entailed a set of open-ended questions that ultimately informed the thematic content of messages and messaging characteristics [30]. Participants were also asked about their perceived barriers and facilitators of regular physical activity. Upon conclusion of this feasibility phase, all messages (n=200) were further refined to incorporate participant feedback and a cognitive framework for behavior change—the capability, opportunity, motivation, and behavior (COM-B) model—and edited and translated by members of the research team.

#### Phase 2: Pilot Testing

**Figure 2:**
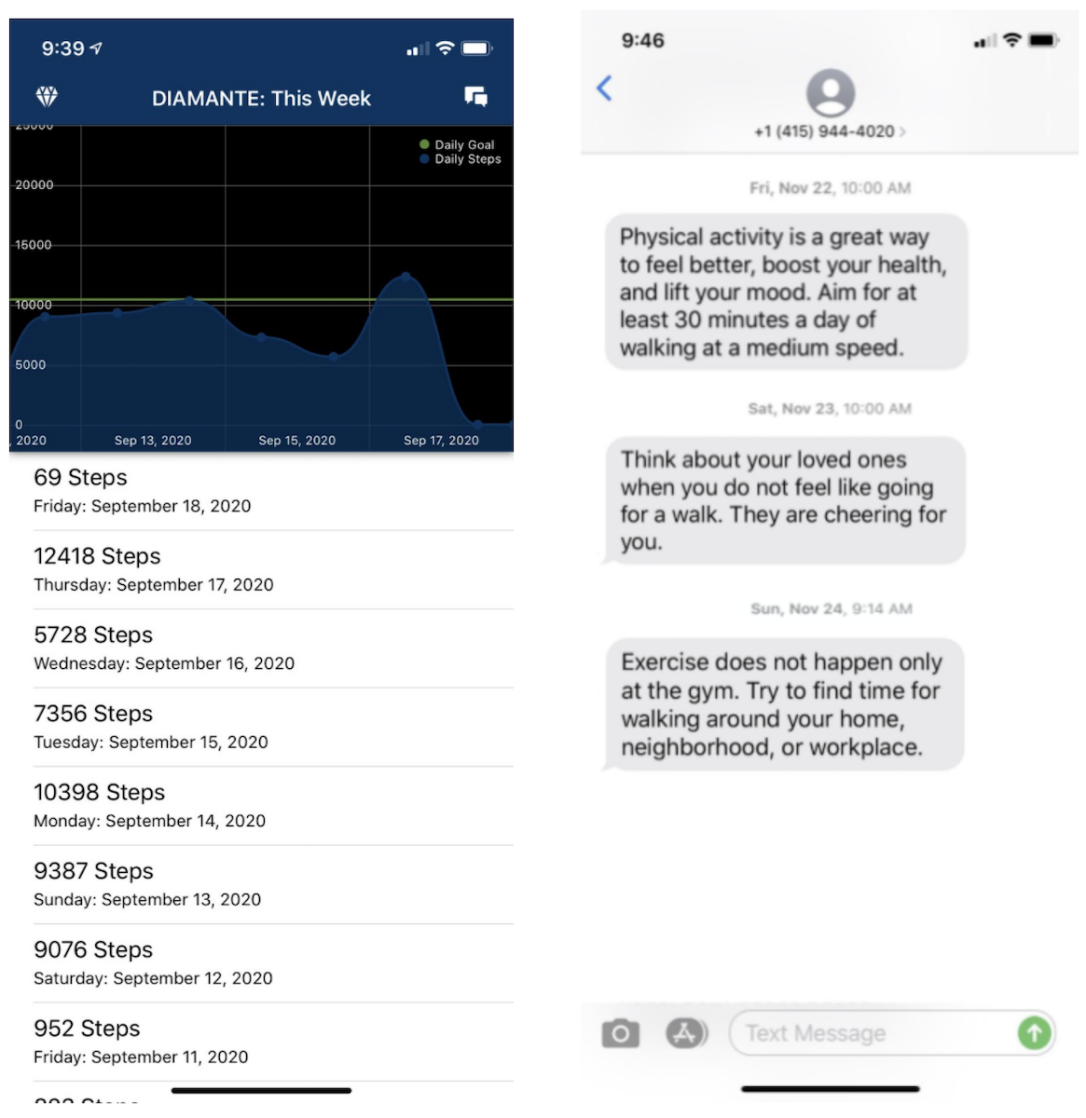
Interface of the smartphone app and intervention text messages in a test user’s texting interface.

This phase took place between January and April 2019. It consisted of a 2-week technology acceptance pilot with English-speaking (n=4) and Spanish-speaking (n=4) patients with comorbid diabetes and depression recruited from the same primary care setting as phase 1. Participants were enrolled on a rolling basis. The pilot entailed testing a subset of messages (n=36), which were updated based on findings from phase 1 (ie, patient feedback from the card sorting activity and interviews). Importantly, these messages were tested within the smartphone app prototype developed by authors AA and CRL and Audacious Software for the DIAMANTE trial [32] (Figure 2). A total of 12 messages from each theoretical category of the full message bank were selected for testing to ensure that the subset (n=36) was representative of our theoretical construct categorization. Moreover, because we wanted to maintain some variability in messaging to evaluate the impact of the adaptive learning algorithm, any messages that received negative feedback in the previous phase were still tested in this phase. The study team members monitored the messages received by the participants. In addition, participants were called by a team member every weekday during business hours at a selected time chosen by the participant to ask them about their experiences with the app and to provide qualitative feedback on the messages. Individualized implementation methods have been recommended for enhancing participant buy-in in maintaining commitment to the intervention once enrolled and minimizing dropout rates in mHealth studies [41,42]. Therefore, in addition to eliciting regular and reliable feedback from participants on the messaging content, we used a daily phone call strategy to enhance engagement with the app and minimize dropouts during pilot testing, particularly among Spanish-speaking participants and those with limited health and digital literacy.

#### Phase 3: Crowdsourcing

This phase took place between February 2019 and March 2019. A subset of 90 messages (30 per category) composed of the 36 messages tested in phase 2 and 54 chosen at random was surveyed among MTurk participants for motivational construct categorization (Table 1). Although a handful of messages (n=4) tested in phases 1 and 2 received predominantly negative participant feedback, we still included these messages in the MTurk sample for variability to assess the impact of the algorithm. As educational attainment and income are correlated [43], we selected participants with high school education or less to inform an intervention targeting low-income users. MTurk participants were asked to read different messages and determine their category (among the 3 overarching theoretical categories: benefit, self-efficacy, and opportunity cues) to the best of their abilities, based on the examples and descriptions provided. Categorization questions were set up as multiple-choice questions with the 3 categories as possible options. The order of the options was not randomized, but the sequence of the questions was randomized. Correct rates (scores) of the English and Spanish categorization questions were calculated for each participant.

**Table 1:** Demographic data of Amazon Mechanical Turk participants (N=134).

Demographic category		Value, n (%)
**Language**		
	English	117 (87.3)
	Spanish	17 (12.7)
**Location**		
	United States	119 (88.8)
	Mexico	4 (3)
	South America	9 (6.7)
	Spain	2 (1.5)

#### Phase 4: Expert Evaluation

This phase occurred in April 2019. After discarding messages (n=10) based on poor performance in phase 3, 80 preselected messages were rated for quality by the study team members using a 5-point Likert-type scale ranging from 1 (poor quality) to 5 (high quality). These ratings were independently performed to reduce bias. In addition, each team member was able to see the entire original set of messages (n=200) and all data from phases 1, 2, and 3.

#### Phase 5: Final Selection

A final bank of messages (n=54) was selected based on team ratings, MTurk scores, and participant feedback. Analysis of the final message selection and final content editing took place from June to August 2019.

## Results

### Phase 0: Message Drafting

We operationalized the constructs of social cognitive theory (ie, self-efficacy and outcome expectancies) [33] into practical cues and tips for physical activity in a subset of messages. In addition, given past research that shows that exposure to proximal positive exercise outcomes increases intrinsic motivation among individuals who report lower levels of past physical activity [34], we framed most messages in terms of proximal outcomes and underscored the positive health-related feelings associated with exercising [35]. Furthermore, we wrote half of the messages with a *gain* framing, emphasizing the benefits of physical activity, and half with a *loss* framing, emphasizing what is avoided by doing physical activity [36]. Health communication research has shown that *gain and loss* framing has the capacity to both increase engagement with message content and change behavior [37,38]. Finally, we wrote messages that followed *individualistic*, *family*, and *peer* orientations to cue participants to increase their current physical activity levels as well as messages that addressed environmental constraints for physical activity [44].

The messages at this stage (n=200) were in a draft form. We aimed to refine the content after gaining a better understanding of the barriers of and beliefs about physical activity of our target population in the subsequent UCD phases, as we anticipated some discrepancy between participants’ identified beliefs and barriers and those we found in the literature. We also sought to identify any silent diabetes- and depression-related barriers for exercise and how well the individualistic, family, and peer orientations resonated with participants.

### Phase 1: Feasibility Testing

Phase 1 consisted of implementing the UCD framework of end user (patient) involvement to obtain qualitative feedback that would allow us to make design decisions. Detailed results of this phase have been published previously [30,40].

#### Card Sorting

Feedback on the existing content consisted of liking messages that allowed for self-reflection, provided concrete ideas for engaging in physical activity, and were highly motivating. In general, participants disliked messages that they perceived as repetitive. Participants also suggested that sticking to highly motivating messages and personalized advice would strengthen the content. Overall, out of the 34 messages evaluated in this phase, 5 (15%) were predominantly liked (by 3 or more participants), 2 (6%) were predominantly considered neutral, and only 1 (3%) message was predominantly disliked.

#### Qualitative Interviews

Key findings revealed 3 main health-related barriers: physical limitations, chronic pain, and depression. We found that the primary facilitators of physical activity included being able to clearly visualize or understand the health-related benefits of engaging in regular physical activity, self-motivation and encouragement to change from a sedentary to an active lifestyle, and offering advice on how to engage in regular physical activity.

#### Theoretical Inclusion

On the basis of participant feedback from the card sorting activity and qualitative interviews, we identified the COM-B model [41] as the optimal theoretical framework for message categorization and conceptualization. According to the COM-B model, behavior change is part of an interacting system involving the key ingredients of capability, opportunity, and motivation [45]. This model lays out a mechanism for the development of effective behavioral health interventions: changing one or more of these components to rearrange the system and minimize the risk of default [45]. Although the COM-B model draws from social cognitive theory, which was the original chosen theoretical base for the messages, we incorporated the COM-B model because it allowed for more optimal linking with our intervention design, as it was a better fit for the opportunity cues to be organized within our message development process.

Accordingly, we reframed our library of draft messages to fit into each of the 3 COM-B model constructs [30]. In targeting the *capability* construct, which is influenced by internal processes that direct behavior, we reconstructed the messages that provided information on the determined benefits of physical activity to highlight physical and social outcome expectations as motivators for physical activity (72/200, 36%). The *opportunity* construct explains the social or environmental factors that allow change to occur. Therefore, we decided to redefine existing messages that addressed environmental constraints for physical activity to emphasize cues for exercise opportunities or suggestions on how to reduce or circumvent these constraints (eg, restructuring routines and planning for social support; 73/200, 36.5%). Finally, for the *motivation* construct, which is demonstrated by the skills and knowledge necessary for change, we used existing messages that targeted self-efficacy (55/200, 27.5%) through tips for building self-management skills and self-beliefs of motivation and confidence to exercise (Table 2).

**Table 2:** Sample of final selected messages by theoretical construct category.

COM-B^a^ construct	Motivational orientation	
	Individual	Social
Capability (benefits of physical activity)	Physical activity is a great way to feel better, boost your health, and lift your mood. Aim for at least 30 minutes a day of walking at a medium speed. Managing your body pain can be hard. Exercise can help! Going for a walk can improve your mood and clear your mind. It may be painful to start walking, but walking often can help with pain relief.	When you are active, you have more energy to spend time with your loved ones. Your loved ones will be proud to see the changes in your health over time. You are important to your loved ones. Being more active can help you take care of them. Being active can improve your mood. This can help you spend quality time with your loved ones.
Opportunity (cues for engaging in physical activity)	Exercise does not happen only at the gym. Try to find time for walking around your home, neighborhood, or workplace. Try not to sit for more than 30 minutes at a time. You can walk around your home for a few minutes. Too many things going on? Take a quick walk to destress and take care of your health. You do not need fancy things to get active. Comfortable shoes and a water bottle should work!	Ask your loved ones for support to be more active. Invite a loved one to go on a walk with you. Use this time to catch up! Change your routine. Go for a walk with loved ones after dinner instead of watching TV. Think about walking as a way for you to visit new places or parks with your loved ones.
Motivation (self-efficacy for physical activity)	Be proud of yourself for making small changes. It is not always easy. You are a strong person. Use that strength to keep trying to reach your walking goal. Believing in yourself is the first step toward reaching your goal. Do not feel bad if you cannot walk very far. Start slow. You will get better!	You can inspire others by being active and staying healthy. Other people will start to see the changes you are making for your health. Think about your loved ones when you do not feel like going for a walk. They are cheering for you. Do not worry if you are not walking as much as other people. Everyone has their own speed.

^a^COM-B: capability, opportunity, motivation, and behavior.

In addition, based on the findings of common constraints for physical activity among our target population, we integrated content that touched on physical symptoms, such as pain, and reframed messages to target the improvement of specific depressive symptoms with the purpose of increasing behavioral activation.

### Phase 2: Pilot Testing

#### Overview

In the initial stage of this pilot, where we evaluated a representative subset of 36 messages (12 for each of the 3 theoretical categories) streamed through the smartphone app prototype, we found that patients tended to not carry their phone or not pay attention to the notification of text messages. However, patients generally started paying more attention to their phones and taking their phones with them more often after a few days into the pilot. In addition, although patients did not always remember receiving text messages when called by the research assistant, all patients reported liking the messages. A total of 75% (6/8) of patients reported feeling indifferent about the timing of the messages.

Furthermore, 38% (3/8) of patients reported not liking the messages in which they were compared with others (eg, other people have the same medical conditions and walked more). Messages about exercising with family members or about the benefits of being healthy to support family members were disliked by 25% (2/8) of participants, who reported not having strong family connections. However, all patients reported that they felt like they started walking more and that they wanted to continue to receive messages past their 2-week trial.

Following the completion of the pilot and based on the negative appraisal among some patients toward messages alluding to family member support, we removed any references to “family,” “peers,” or “friends” and replaced them with a blanket “loved ones” phrase. In the same vein, we decided to merge the *family* and *peer* orientations as *social* and therefore reclassify the messages as either *individualistic* or *social*.

#### Editing

Message editing was performed by the first author. The purpose of editing was the overall quality assurance of all existing messages. The first round of edits focused on identifying and correcting grammar, spelling, and punctuation errors as well as simplifying syntax to a lower reading level. All messages were adapted to a reading level of sixth grade or below (average=3.5). A web-based readability tool [46] was used to measure the reading level of each message using the Flesch-Kincaid grade level formula. Messages with scores higher than 6 were edited until they scored lower than the sixth-grade cutoff. A second round of edits focused on rewording to make the messages more concise to improve the experience of patients reading them. As the number of characters that can fit in a standard SMS is 160, messages with more than 160 characters were edited and reduced to fit this parameter. However, as the research staff had identified patient preferences for shorter messages in the previous phases, most messages were already under 140 characters.

#### Translation From English Into Spanish

The English text messages were translated into Spanish by the bilingual research staff. The translated messages were also reviewed by a native Spanish speaker fluent in English. In general, all translations were performed following a culturally sensitive communication standard [47]. We adapted syntax, lexical content, and idiosyncratic phrases that have been identified as key cultural aspects of translation in previous text messaging intervention research [47]. This means that the messages were not translated literally; rather, they were adapted and reframed to consider the ethnic diversity and other important cultural characteristics of one of the target populations of the DIAMANTE study—Spanish-speaking immigrants from Latin American countries. As such, neutral Spanish [48] words were used to the extent possible, and formal Spanish was used throughout (ie, using “usted” and its derivations in second person pronouns). A translation key was developed to standardize commonly translated phrases or words (Multimedia Appendix 1). During editing, any changes to English messages were simultaneously applied to the translated Spanish messages. Similar measures to reduce the literacy level of English content were applied to the Spanish content.

### Phase 3: Crowdsourcing

In this phase, we submitted a representative sample of messages (n=90, 30 for each theoretical category) to the MTurk participants to test and validate the theoretical categorization. MTurk testing enabled us to ensure that the messages had been tagged in the right motivational categories and, therefore, refine the content and select the best ones. Messages were placed in the right category at an average of 58% (SD 0.21%) of the time. The MTurk respondents also rated messages for their overall quality from 1 to 5. The highest-rated messages were those in the *benefit* category. A total of 70% (63/90) of the messages were put in the right category at least 50% of the time. Furthermore, 11% (10/90) of the messages yielded a mean percent correct categorization rate of 40% or less. These messages were discarded from subsequent evaluations because of their thematic ambiguity. Participants completed the survey in an average of 6.5 minutes (Table 3).

**Table 3:** Amazon Mechanical Turk classification results of messages by theoretical category (N=30).

Theoretical category	Correct classification, mean n (%)
Benefit	21 (70)
Self-belief	16 (53)
Opportunity	15 (50)

A challenge with using MTurk was to recruit a larger sample of Spanish-speaking workers to test the theoretical categorization of the messages. Otherwise, the task requirements of our categorization tests were easily implemented at the interface of the platform. However, as we found that the qualitative feedback about the message content from the testers was either incomplete or not helpful (ie, not reflective of the needs of our target population), we decided to discontinue this task after the second round of testing.

### Phase 4: Expert Evaluation

To assess the content adequacy of the messages, all coauthors independently rated the high-performing messages of phase 3 (n=80) for subjective quality using a scale of 1 to 5. The coauthors gave the messages an average rating of 3.6 (SD 0.59) and reached a quality rating consensus for a given message only 3% (2/80) of the time. A total of 60% (48/80) of the messages received a rating of 3.5 and higher. Quantitative ratings and qualitative feedback were collected from each coauthor and analyzed in aggregate.

### Phase 5: Final Selection

Feedback from all 4 phases was combined to select the best messages from the preselected subset from phase 4 (n=80). Messages that compared patients with others with the same medical conditions, which were predominantly disliked by participants in phases 1 and 2, were discarded (4/80, 5%). Of the remaining messages, those that were both sorted into the correct theoretical categories at least 50% of the time during phase 3 and rated at least 3.5 or higher in quality during phase 4 were considered as the clearest and highest impact content to include in the final text message bank. This combined feedback resulted in a final bank of 54 messages, with 18 messages per motivational category.

## Discussion

### Principal Findings

Using a theory-based UCD framework, which consists of an iterative development process blending UCD methods with crowdsourcing and expert input, we produced a library of 54 motivational messages for a physical activity SMS intervention for low-income minority patients with comorbid diabetes and depression over a 2-year period. The development process comprised 5 phases: feasibility testing, pilot testing, crowdsourcing, expert evaluation, and final selection.

### Lessons Learned

Patient feedback gathered in the UCD phases (1 and 2) was overwhelmingly positive: most patients reported no preference for the timing of message delivery, were receptive to receiving motivational text messages, and reported wanting to continue receiving messages upon conclusion of the pilot testing. However, user feedback is prone to response bias, and it is possible that such positive responses were because of patients not wanting to disappoint the study investigators. Findings from the UCD phases were especially helpful for discarding unappealing messages and editing thematic aspects of messages that did not resonate well with patients. For example, after phase 2, we rephrased any references to peer or familial relations to a broader social denomination (*loved ones*) based on the finding that participants who reported not having a family disliked messages that alluded to family support. The number of content changes we incorporated over 2 rounds of UCD testing underscores the importance of taking a user-centered approach to the development of a text messaging intervention. Results from the MTurk testing phase 3 showed that this platform is a relatively inexpensive, accessible, and rapid source of data for the validation of message classification. Thus, we learned that crowdsourcing methods are valuable for certain aspects of the design process in which a larger group of participants is desired or when seeking specific feedback that is not about engagement with content, such as for determining the correct thematic categorization of messages. Therefore, it is valuable to leverage crowdsourcing platforms as a more accessible and inexpensive source of feedback. Finally, we found that expert input and review (phase 4) rendered cohesiveness and reliability to the design process, given that this stage consisted of making independent ratings of the final set of messages to achieve consensus on the best ones. This was particularly important when it came to translating theory and evidence into succinct and engaging yet actionable and motivational messaging.

### Strengths and Limitations

The strengths of this study include the multistaged and evidence-based development of cogent messaging that is grounded in the available scientific best practices, health behavior change and user engagement theories, and feedback from target users. This process allowed us to design content to match and adapt to the relevant needs of our patient population. As such, we wrote all messages at a sixth-grade or lower reading level, took cultural and linguistic appropriateness into account during editing and translation, and tested for acceptability and clarity among target patients. However, this study has some limitations. For instance, the sample sizes for the UCD phases were small and from one geographic location. However, the purpose of qualitative user research is not to produce findings that can be generalized to other populations but rather to generate data about attitudes or behaviors based on direct observation. Indeed, the UCD phases in our study were used to assess the acceptability and usability of a set of motivational text messages for use in a future physical activity intervention study tailored to a diverse clinical sample of low-income patients with diabetes and depression. A second limitation of our study is that although we did not collect demographic information beyond language and the country of residence, MTurk respondents are not likely to be representative of our target population. Thus, despite the aforementioned benefits of sampling from this crowdsourcing platform, findings from phase 4 were interpreted with caution, only used for theoretical category validation, and not used to inform subsequent content changes. A third limitation is that MTurk respondents did not receive the full set of draft messages (n=200) in the theoretical category validation task, which means that we were not able to collect categorization ratings for every message. However, we had raters assess only a subset of messages (n=90) as a measure to reduce the overall burden of the task and not overwhelm the respondents. We also noted messages with motivational construct ambiguity that were not tested in MTurk during the internal expert review phase and later discarded them from the final selection. A fourth limitation is that the MTurk categorization responses are subject to method bias as a function of low motivation; that is, participants may have not been willing or able to expend the required amount of cognitive effort and thus were less thorough in making accurate response selections. Online recruitment methods such as MTurk have been criticized as prone to yield samples full of low effort respondents [49]. However, we operationalized measures to reduce such bias in this content validation phase, including providing clear and precise definitions of the 3 motivational categories and providing a *trial* run during the onboarding stage for MTurk participants.

### Future Directions

This paper describes in detail the process for designing and validating the content of an mHealth intervention that utilizes an adaptive learning algorithm to deliver optimized and tailored motivational text messages that promote physical activity. Previous studies have utilized similar methods to inform health text messaging development, such as message drafting based on behavior change theory [16,23] and public health guidelines [18], survey responses of participants’ preferences [23], focus groups and expert evaluation [18], and end user ratings of content understanding and appeal [16]. However, the process described herein is unique in that it integrates various design methods previously reported in literature and incorporates crowdsourcing via MTurk as an alternative content pretesting method. This is important because beyond a focus on usability, engagement, and implementation, alternative methods for intervention development in general and text messaging content in particular are a largely untapped area of inquiry in the field of mHealth. This paper thus helps advance mHealth research addressing intervention design processes, including content pretesting methods, which are not commonly detailed in the literature [17]. However, there continues to be a need for research on text messaging intervention design methodology, especially that which explores how to best integrate behavioral health theory in messaging content. There is also a need to continue evaluating new empirical models of content testing, including those that leverage crowdsourcing and online recruitment methods such as Amazon’s MTurk and Facebook.

### Conclusions

We present a novel, integrative framework for combining UCD, expert input, and crowdsourcing to determine, create, and improve the content of text messages of a smartphone app to increase physical activity in low-income, English- and Spanish-speaking patients with depression and diabetes. This provides a research-based design approach for future developers of health text messaging interventions. This framework can potentially be extended to mHealth programs targeting other vulnerable populations and health behaviors.

## Appendix

Multimedia Appendix 1 Translation key.

## Abbreviations

COM-B: capability, opportunity, motivation, and behavior
DIAMANTE: Diabetes and Mental Health Adaptive Notification Tracking and Evaluation
mHealth: mobile health
MTurk: Amazon Mechanical Turk
UCD: user-centered design
