# Supplementary material for: Developing Messaging Content for a Physical Activity Smartphone App Tailored to Low-Income Patients: User-Centered Design and Crowdsourcing Approach
Source: JMIR Mhealth Uhealth. 2021 May 19;9(5):e21177. doi: 10.2196/21177 (PMC8173396; doi:10.2196/21177)
Supplement: Multimedia Appendix 1 [file mhealth_v9i5e21177_app1.docx]

## Appendix A. Translation Key

| English | Spanish |
| --- | --- |
| physical activity | actividad física / ejercicio |
| mood | estado de ánimo |
| (to) lose weight | bajar de peso |
| being active | estar activo/a |
| walking often | caminar seguido / caminar con frecuencia |
| walking regularly | caminar con regularidad |
| long walk | caminata larga |
| quick walk | caminata rápida |
| going for a walk | salir a caminar |
